# Selected serum cytokines and vitamin D levels as potential prognostic markers of acute ischemic stroke

**DOI:** 10.1371/journal.pone.0299631

**Published:** 2024-06-13

**Authors:** Nirmali Samarakoon, Thashi Chang, Vimukthi Gunasekara, Praneeth Ratnayake, Rasika Jayatillake, Preethi Udagama

**Affiliations:** 1 Faculty of Science, Department of Zoology and Environment Sciences, University of Colombo, Colombo, Sri Lanka; 2 Faculty of Medicine, Department of Clinical Medicine, University of Colombo, Colombo, Sri Lanka; 3 Faculty of Science, Department of Statistics, University of Colombo, Colombo, Sri Lanka; National Institutes of Health, UNITED STATES

## Abstract

Inflammation-derived oxidative stress is postulated to contribute to neuronal damage leading to poor clinical outcomes in Acute Ischemic Stroke (AIS). We aimed to investigate the association between serum levels of selected cytokines (IL-1β, IFN-γ, IL-4), and vitamin D in ischemic stroke progression, and their accuracy in predicting AIS prognosis, among Sri Lankans. We compared 60 AIS patients admitted in 4 phases post-stroke onset (<6 h; 6–24 h; 24–48 h; 48–96 h; n = 15/phase), with 15 age- and sex-matched controls. The 30-day functional outcome (FO) was assessed using the modified Rankin Scale (mRS). Serum cytokine and vitamin D levels were quantified using sandwich ELISAs, and competitive ELISA, respectively. The CombiROC web tool established optimal prognostic biomarker combinations. Serum IL-1β and IFN-γ were elevated in all four phases following stroke onset while IL-4 was elevated exclusively in the recovery phase (48–96 h) (p<0.05). Th1 bias polarization of the Th1:Th2 cytokine (IFN-γ:IL-4) ratio occurred with AIS progression while a Th2 bias occurred during AIS recovery (p<0.05). Lower serum IL-1β and higher IL-4 levels were associated with a good FO (p<0.05), while lower Vitamin D levels were related to a poor FO (p = 0.001). The triple-biomarker panel, IL-4- IFN-γ -Vit D, accurately predicted AIS prognosis (sensitivity = 100%, specificity = 91.9%, area under the curve = 0.98). Serum immunologic mediators IFN-γ, IL-4, and vitamin D may be useful biomarkers of AIS prognosis and may serve as therapeutic targets in improving stroke outcomes. Vitamin D supplementation may improve the prognosis of AIS patients. Furthermore, binary logistic model fitted for FO indicated Th1:Th2 cytokine ratio (IFN-γ:IL-4), vitamin D status, history of stroke, and ischemic heart disease as significant predictors of AIS prognosis.

## Introduction

The outcome of a wide range of neurological disorders including stroke is linked to neuroinflammation in the central nervous system (CNS). In Acute Ischemic Stroke (AIS), this Inflammation leads to oxidative stress, which is postulated to contribute to neuronal damage, leading to poor clinical outcomes [1]. AIS-associated neuroinflammation is mediated by activated brain-residing astrocytes, microglial cells, and infiltrating T lymphocytes [2]. In AIS, abnormalities in peripheral immune functions, such as changes in lymphocyte subpopulations in blood, deviation of T-lymphocyte subsets, damage to the blood-brain barrier (BBB), and increased cytokine levels have been reported [3]. This creates a cytokine storm leading to organ dysfunction and, thus, the potential for improving outcomes by addressing the immune system dysregulation in AIS [4].

Cytokines IL-1β, IFN- γ, and IL-4 are key players post-AIS. After an AIS, IL-1 β triggers adhesion molecules and proinflammatory cytokines, activating microglia and astrocytes, and has been reported to correlate with poor functional outcomes [5,6]. T lymphocytes and Th1 cell-derived IFN-γ contribute to tissue damage, microvascular dysfunction [7] and M1 microglial polarization, promoting a proinflammatory response [8]. In contrast, IL-4 may alleviate AIS injuries through enhanced angiogenesis and exosome release with miRNA-26a. Additionally, IL-4 exerts neuroprotective effects by stimulating IL-4/STAT6 signaling and inhibiting proinflammatory cytokines, playing a vital role in repairing brain damage, suppressing post-stroke inflammation, and inducing neurotrophic factors in astrocytes [9,10].

Vitamin D serves as a crucial signaling molecule in both innate and adaptive immune systems, primarily acting as an anti-inflammatory agent [11]. Vitamin D deficiency is often underdiagnosed and untreated, making it a significant nutritional deficiency worldwide [12]. Lack of vitamin D has been implicated with a higher risk of vascular diseases including ischemic stroke [13]. Between 2000 and 2002, a 15.7% global prevalence of insufficient serum 25(OH)D levels (<30 nmol/L) has been reported [12]. Recent research has identified associations between 25(OH)D deficiency and functional impairments, as well as unfavorable survival outcomes in AIS [14].

Despite the recent surge of therapeutic options including reperfusion therapy, stroke remains a leading cause of adult mortality and disability [15,16]. The identification of novel therapeutic targets and the ability to predict early prognostic endpoints is likely to mitigate the burden of stroke. We aimed to characterize the role of pro- and anti-inflammatory serum cytokines, and vitamin D in the progression of AIS, and to determine the use of multi-biomarker panels as prognostic tools in AIS.

## Materials and methods

### Study design and participants

The study protocol was approved by the Ethics Review Committee, Faculty of Graduate Studies, University of Colombo, Sri Lanka (ERC/FGS/2021/025). This prospective case-control study is reported in accordance with the STARD guidelines for diagnostic and prognostic studies [17].

The sample size was determined by the formula (N/r = r+1x SD^2^[Z_β_ + Z_α/2_]^2^/d^2^) [18]. The sample size thus obtained was N = 120. Due to limited funds, this was pragmatically reduced to n = 60 AIS patients.

Patients who were admitted to the National Hospital of Sri Lanka (NHSL), Colombo, at four phases of post-stroke onset (<6 h, 6–24 h, 24–48 h, 48–96 h; n = 15/phase; total n = 60) were recruited for the study from November 2021 to April 2022. Age and sex-matched healthy adult volunteers (n = 15) with the same ethnic origin and area of residence served as normal, healthy controls. Diagnosis of AIS was made based on well-established clinical criteria [20]. Exclusion criteria included patients presenting with evidence of acute infections, fever, inflammatory, hematological, or autoimmune diseases, as well as those with liver, kidney or heart failure, cancer, and cerebral hemorrhage. All patients and controls were recruited for the study following voluntary, written, informed consent.

On admission to hospital, patients underwent clinical evaluation to determine the stroke severity using the National Institute of Health Stroke Scale (NIHSS). The functional outcome (FO) 30 days following AIS onset was assessed using the modified Rankin Scale (mRS). Socio-demographic information was collected through an interviewer-administered questionnaire. The participants were recruited to the study based on consecutive sampling (total enumerative sampling). Each AIS patient in order of admission to the hospital, meeting the inclusion criteria were selected until the required sample size was achieved in each of the post-stroke phases [19].

### Detection of serum levels of cytokines and vitamin D

#### Serum cytokines

Serum levels of cytokines IL-1β, IFN-γ, and IL-4 (BD OptEIATM Set, Human IL-1β, Catalog Number: 557953; Human IFN-γ, Catalog Number: 55142; Human IL-4, Catalog Number: 55194) were quantified using specific human sandwich Enzyme Linked Immunosorbent Assay (ELISA) kits, in accordance with the instructions provided by the manufacturer (Becton & Dickinson OptEIA™, USA). Monoclonal antibodies were used for capture and detection that selectively targeted and bound the specific cytokines as antigens present in the serum samples.

#### Serum vitamin D

Serum levels of vitamin D (25[OH]D) were quantified using the total 25-HydroxyD competitive ELISA kit, in accordance with the manufacturer’s protocol (Global Diagnostics™, Belgium, Catalog number 7119). Vitamin D was detected as antigen in serum using capture monoclonal antibodies specifically designed against vitamin D.

25(OH)D concentration in the bloodstream is higher than other vitamin D metabolites. Additionally, in contrast to vitamin D, the bulk of 25(OH)D in the body is found in blood, with limited distribution into less accessible storage sites such as fat. Assessing its concentration in the blood provides the best reflection of vitamin D status due to its longer half-life in blood, and the first–order kinetics, wherein the rate of 25(OH) D production is dependent on vitamin D levels [20].

#### CombiROC curve analysis

The efficacy of the prognostic markers of AIS was assessed using receiver operating characteristic (ROC) curves. To evaluate the predictive value of the tested serum cytokines and vitamin D marker combinations, a combinatorial analysis of multiple biomarker signatures was performed using the freely accessible CombiROC web tool (http://CombiROC.eu) [21]. A test cutoff value was derived as the control mean ± 2SD.

Furthermore, to obtain a predictive model based on the selected combination of the CombiROC outcome, a logistic regression model was fitted using backward stepwise method in “step AIC” function in R.

### Statistical analyses

SPSS version 26.0 (IBM Corporation, New York, USA), and Prism Version 9.0 (GraphPad Software, Inc., San Diego, California, USA) software packages were used to statistically analyze the generated data. Prior to analysis, the data were assessed for normality distribution. Continuous variables that exhibited non-normal distribution were compared using non–parametric tests. The Kruskal-Wallis H test was applied to examine the variation of the analytes across distinct groups of stroke patients. The Mann–Whitney U test compared levels of each tested analyte in good/bad functional outcomes based on the 30–day FO. Chi square test was performed to investigate the association between risk factors and the 30-day functional outcome of AIS.

The calculated odds ratio (OR) quantified the odds of a poor prognosis associated with risk factors of stroke in AIS patients.

Univariate binary logistic regression was performed to explore the independent effects of serum levels of vitamin D and cytokines (IFN-γ, IL4, IL-1β) as analytes, and the effect of time of admission after stroke onset, on the 30-day functional outcome of the AIS patients.

Independent variables (i.e. Th1:Th2 cytokine ratio [IFN-γ:IL-4], Vitamin D, gender, time of stroke onset, age, history of stoke, history of transient ischemic attack, high blood pressure, ischemic heart disease, cardiac structural abnormalities, atrial fibrillation, diabetes mellitus, dyslipidemia, family history of stroke, smoking, alcohol, Oxfordshire stroke classification, and severity of stroke were investigated to build an algorithm to predict the FO. Initially, separate univariate binary logistic regression for each independent variable were conducted at 20% significance level to identify the variables that have significant association with the 30-day FO of AIS patients. Using these identified variables, multivariable logistic model was fitted using ‘*glm*’ function and backward stepwise model selection method using the “stepAIC” under the “MASS” package [22]. The best model was selected with the lowest Akike information criteria (AIC) value [23]. The package “*dplyr*” was used to handle the data [24], while the analysis was carried out in the R statistical software [25].

Statistical significance was set at p<0.05.

## Results

### Patient characteristics

The demographic and clinical characteristics of the participants, detailed in supplementary materials (S1 Table), revealed no significant differences among the subject groups (p>0.05).

### Levels of selected serum cytokines and vitamin D of AIS patients

**Fig 1:**
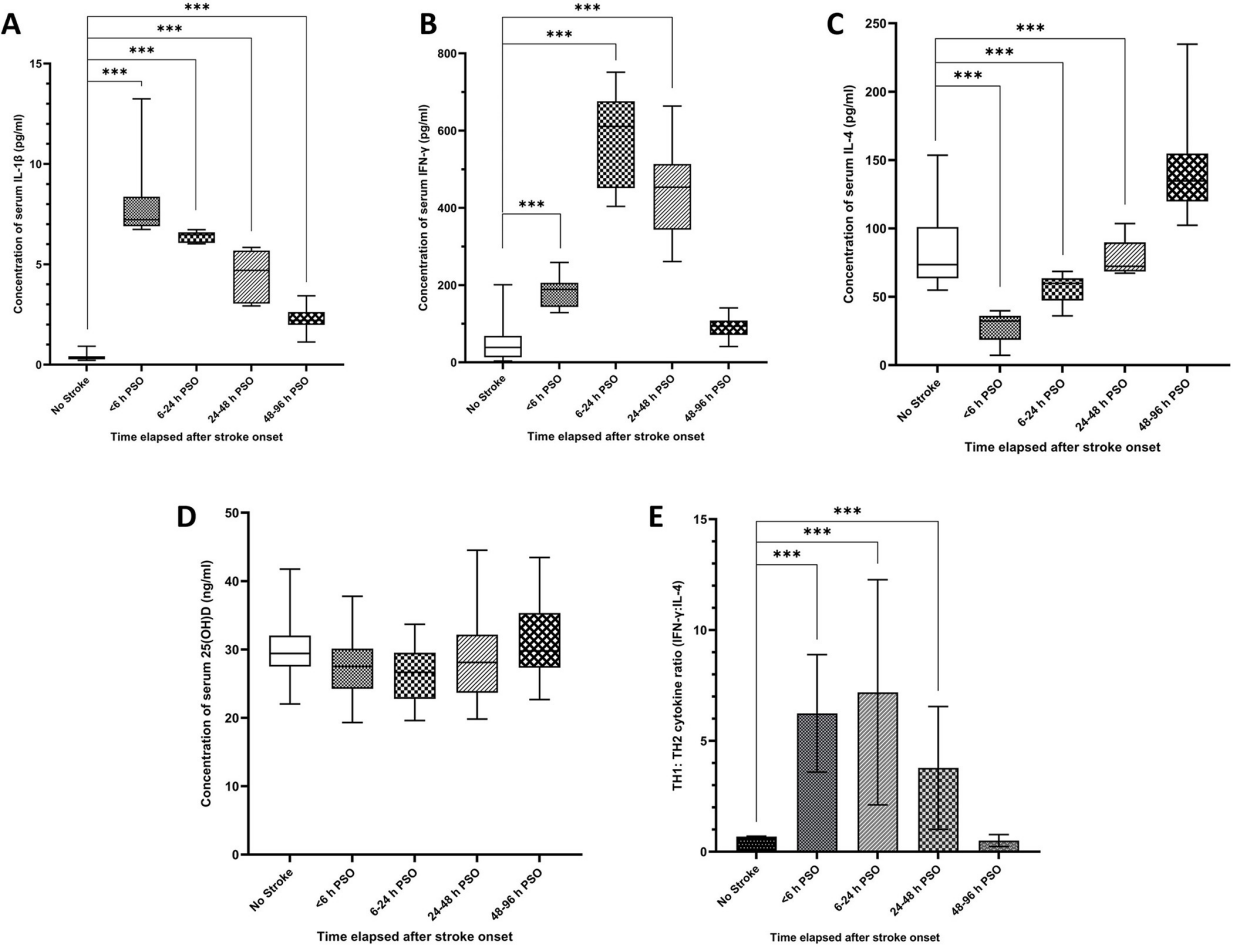
Concentration of serum immune mediators and the Th1:Th2 cytokine ratio (IFN-γ: IL-4) in AIS patients at different time points following stroke onset, compared to healthy controls. Levels of serum A) IL-1β, B) IFN-γ, C) IL-4, D) Vitamin D and E) Th1:Th2 cytokine ratio (IFN-γ: IL-4) of AIS patients. Group 1- patients admitted within <6 h, Group 2- within 6–24 h, Group 3-within 24–48 h, and Group 4- within 48–96 hours, post stroke onset (***p<0.001).

In comparison to healthy controls, a significant and sequential reduction in the serum IL-1β levels with the stages of stroke progression (<6 h > 6–24 h> 24–48 hours> 48–96 hours) was observed in AIS patients (P<0.001) (Fig 1A). A significant peak in the serum IFN-γ concentration was evident in AIS patients admitted 6–24 h after stroke onset, compared to healthy controls (P<0.001) (Fig 1B). Conversely, a sequential increase in the serum IL-4 concentration was observed with the stages of AIS progression (P<0.05) (Fig 1C). Nonetheless, no significant differences were observed in the mean serum vitamin D concentrations, either among the test groups and the control group, or the four test groups (P>0.05) (Fig 1D).

### Th1:Th2 cytokine ratio

The ratio of serum concentrations of Th1:Th2 (T helper subset 1: T helper subset 2) cytokines, represented by IFN-γ:IL-4 showed a clear Th1 polarization with AIS progression while a Th2 bias was evident during AIS recovery (p<0.05) (Fig 1E).

### CombiROC performance analyses for optimal marker combinations

The “gold combinations” IL-4- IFN-γ (Area under the curve [AUC] = 0.984, Sensitivity [SE] = 100%, Specificity [SP] = 91.9%), IL-4-Vit D (AUC = 0.984, SE = 100%, SP = 91.9%), and IL-4- IFN-γ -Vit D (AUC = 0.982, SE = 100%, SP = 91.9%) showed high values for all metrics including AUC, SE, and SP (S2 Table). Importantly, the combo of IL-4, IFN-γ, and Vitamin D may be selected as the best biomarker triplet as a *de novo* prognostic panel for AIS.

A ten–fold cross-validation (CV) was used to obtain a reliable estimate of the overall performance of the prognostic marker panel. A permutation test was conducted to overcome the over–optimistic results obtained by the CV, where the distribution of the data set was reassumed by resampling the observed data. The results obtained (S3 Table) suggested that the panel was a fit as the overall accuracy (ACC), SE, and SP were least affected by the imposed likelihood (S3 Table and S1 Fig).

A predictive model based on the best triplet biomarker combo of IFN-γ, IL-4 and Vitamin D, was fitted using backward stepwise method in “stepAIC” function. This resulted in the following model (Eq 1) with an efficiency of 96%:

$$\log\left(\frac{\hat{p}}{1-\hat{p}}\right)=(5.82470)-0.02665(IL4)-0.04681(IFN-Gamma)$$ (1)

This model indicates variables IFN-γ and IL-4 as best predictors for AIS.

### Association of the functional outcome with risk factors of AIS

Previous history of stroke, ischemic heart disease and vitamin D deficiency were associated with a significant 30-day poor FO (Table 1). Of these, the former two health states posed significant relative risk for a poor AIS prognosis, whereas vitamin D deficiency conferred a 25.9-fold (p<0.001) greater chance of a poor prognosis.

**Table 1:** Association of the modified Rankin scale 30-day functional outcome and risk factors of acute ischemic stroke.

Risk factor	mRS outcome		Chi-square	P–value	OR	Relative risk (RR)	95% CI	
	Good	Poor					Lower Bound	Upper Bound
Previous history of stroke								
Yes	5 (8.33%)	10 (16.67%)	6.79	0.01**	0.2	3.22	1.26	8.23
No	32 (53.33%)	13 (21.67%)						
Presence of ischemic heart disease								
Yes	5 (8.33%)	8 (13.33%)	3.78	0.05*	0.29	2.57	0.96	6.92
No	32 (53.33%)	15 (25%)						
Vitamin D status								
Sufficiency	20 (33.33%)	1 (1.66%)	11.69	0.001***	25.9	1.77	1.29	2.41
Insufficiency	17 (28.33%)	22 (36.66%)						

^a^ modified Rankin scale (mRS) score: Good outcome = 0–2; poor outcome = 3–6.

### Effects of analytes and time of hospital admission post stroke onset on the 30-day functional outcome

From the univariate binary logistic regression carried out to test for the effects of cytokines and vitamin D on the 30-day functional outcome assessed on the mRS (Table 2A), IL-1β showed a significant negative value (p<0.021) indicating that an increase in IL-1β resulted in a poor FO. Conversely, vitamin D confirmed a significant positive effect (p<0.007) on the FO, where increasing Vitamin D levels tend to improve the FO. Conversely, the time of hospital admission post-stroke onset had no effect on the 30-day FO (Table 2B).

**Table 2:** A. Effects of different cytokines (IFN-γ, IL-4, IL-1β) and vitamin D on the 30-day functional outcome (mRS). B. Effect of time of hospital admission post stroke onset on the 30-day functional outcome (mRS).

**Variable**	Level^a^	**Estimate**	**Z-Value**	**Odd Ratio**	**95% CI**		**p-value**
					2.5%	97.5%	
**Intercept**		0.1335	0.1335	1.1428	0.4102	0.796	3.2595
**Variable**	**Estimate**	**Odds Ratio**	**95% CI**			**Z-Value**	**p-Value**
			2.5%	97.5%			
**IL-1β**	-0.3080	0.7348	0.5504		0.9391	-2.291	0.021*
**IL-4**	0.0136	1.0137	1.0007		1.0294	1.920	0.055 .
**IFN-γ**	-0.0015	0.9984	0.9959		1.0009	-1.205	0.228
**Vitamin D**	0.1819	1.1995	1.0643		1.3898	2.698	0.007 **
**Time of hospital admission post stroke onset**	1	-0.5390	-0.730	0.5833	0.1317	0.466	2.4619
	2	0.8781	1.125	2.4063	0.5367	0.260	12.020
	3	1.2528	0.8274	3.5000	0.7348	0.130	20.304

^a^ Level 1:-24 hrs; Level 2: 24–48 hrs; Level 3: 48–96 hrs, post stroke onset.

mRS—modified Rankin scale: Good outcome = 0–2; poor outcome = 3–6.

### Model to predict the 30-day functional outcome

The stepwise binary logistic regression model fitted for FO of AIS (S1 Appendix) established that Th1:Th2 cytokine ratio (IFN-γ:IL-4), vitamin D status, history of stroke, and ischemic heart disease (Eq 2) can be used to predict the 30-day functional outcome of AIS patients at an efficiency of 80% (AIC– 61.66, AUC– 86.84%) (Table 3).

Eq 2. FO prediction algorithm:

$$\log\left(\frac{\hat{p}}{1-\hat{p}}\right)=(-5.07016)-0.12888(Cytokine\;Ratio)+0.26867(Vitamin\;D)-1.88874(History\;of\;Stroke)-2.81101(Ischaemic\;Heart\;Disease)$$ (2)

Where, $\hat{p}$ is the estimate of P (positive functional outcome)

**Table 3:** Estimates and odds ratios of the parameters of the model selected from backward “stepAIC” method.

Variable	Estimate	Odds Ratio	95% CI		Z-Value	p-Value
			2.5%	97.5%		
**(Intercept)**	-5.07016	0.0062	0.000047	0.37893	-2.253	0.024*
**Th1:Th2 cytokine ratio**	-0.12888	0.87908	0.7493	1.0165	-1.697	0.089 .
**Vitamin D**	0.26867	1.3082	1.1267	1.5846	3.146	0.002 **
**History of stroke**	-1.88874	0.1513	0.0235	0.7390	-2.197	0.028 *
**Ischaemic heart disease**	-2.81101	0.0601	0.0054	0.4072	-2.615	0.009 **

## Discussion

Inflammation plays a complex and dual role in the pathogenesis of ischemic stroke. While it can exacerbate secondary brain injury during the acute stage of stroke, it also plays a crucial role in supporting recovery processes following the stroke. This intricate balance between harmful and beneficial effects is regulated by various inflammatory cells, cytokines, and inflammatory chemicals [26].

A significant Th1 polarization in the Th1:Th2 (IFN-γ:IL-4) cytokine ratio during AIS progression, with a Th2 bias during AIS recovery identified in our study suggest that subtle changes in the peripheral immune micro-environment play a crucial role in the pathogenesis and outcome of AIS. T cells play a significant role in the process of neuronal injury and repair. Different T cell subsets have been speculated to have different effects on ischemic brain tissue depending on their functional characteristics [1]. Our study confirms previous reports on the peripheral cytokine profile regarding IL-1β, IFN-γ, and IL-4 that demarcated an upsurge of IL-1β and IFN-γ during the onset and progression stages of AIS. These elevated cytokine levels may be indicative of the infiltration of T cells into the ischemic brain tissue and the efflux of inflammatory mediators through the damaged BBB [27]. The increased IFN-γ levels in AIS progression we observed presumably hastened tissue damage, neuronal autophagy, and thrombosis. Conversely, the polarization towards an anti-inflammatory Th2 phenotype during the recovery stage of AIS is suggestive of immunosuppressive and reparative processes, underscoring the potential for targeted pharmacological interventions to modulate this cytokine, presenting an exciting avenue for enhancing post-AIS recovery outcomes through therapeutic manipulation. A reference range for the serum levels of IL-1β, IFN-γ, and IL-4 remains unclear due to potential fluctuations of the measured cytokine levels influenced by time of the day during collection, fasting, food digestion, physical activity, stress, arterial-venous differences, sampling methods, storage conditions, and the sensitivity of the immunodiagnostic technique used [28]. Additionally, findings from globally conducted studies may not be directly relevant in a Sri Lankan context as the individual genetic profiles particularly in pathways such as the NF-κB that plays a vital role in cytokine synthesis, can differ due to the diverse and varying environmental factors, dietary habits, and hereditary factors specific to a Sri Lankan population [29].

Consistent with previous studies, we found that lower serum concentrations of IL-1β and higher concentrations of IL-4 levels to be associated with a good 30-day functional outcome of AIS which may be attributed to the pro-inflammatory and anti-inflammatory properties of the two cytokines, respectively [30–32]. The available evidence on the time course of post–stroke inflammation is both limited and ambiguous. However, there is a growing body of evidence suggesting an association between neuroinflammation and functional outcomes [33]. Following an ischemic stroke, cerebral injury occurs initially due to ischemia and subsequently due to neuroinflammation that occurs during ischemia and reperfusion. The phases in our study were selected to represent the hyperacute and acute phases in the first 24 hours post-stroke in which ischemic injury occurs, and subsequently the reperfusion and repair phases in which there is an interplay between pro- and anti-inflammatory mediators [1]. The inflammatory response is a dynamic and coordinated process involving various cells, signaling molecules, and tissue repair mechanisms. However, these phases are not strictly delineated, and there can be overlaps between them.

MRI scans from patients with ischemic stroke reveal the development of small vessel disease and white matter damage [34]. Patients with adequate vitamin D supplementation demonstrate lowered blood pressure, vasodilation and restoration of blood flow to neurons [35]. The activation of insulin-like growth factor 1 (IGF-1) by 1,25(OH)2D3 plays a key role in influencing the regulation of axon and dendrite degeneration and exert antithrombotic properties via plasminogen activation [36]. Many studies, however, indicate that normal vitamin D levels activate neuroprotective mechanisms that protect against BBB dysfunction associated with a developing stroke focus [37]. Furthermore, recent research suggests that vitamin D protects endothelial cells from BBB disruption in ischemic stroke [38].

A 10.5 likelihood of bearing a poor outcome with each nanogram per milliliter decrease in vitamin D levels has been reported previously [39]. This was substantiated in our study with an exceedingly high odds ratio of 25.9, thereby establishing a significantly strong likelihood for individuals with vitamin D insufficiency to experience poor post-stroke outcomes. Vitamin D deficiency has been reported in approximately 58.5% of the population in Sri Lanka despite an abundance of sunlight [40]. A protective impact of vitamin D on functional outcomes and death following ischemic stroke has been shown in several observational studies [41,42]. Low 25(OH) D levels were shown to alter lymphocytic and macrophage activity in atherosclerotic plaques inducing chronic arterial wall inflammation, morphologic brain alterations and motor deficits, rapid bone resorption, lower bone mineral density, endothelial dysfunction, pro-atherosclerotic variations in vascular smooth muscle cells, and an increase in macrophage foam cell production in stroke patients acting as a true neuroactive steroid [43]. However, additional studies are required to elucidate the intricacies in the brain cytokine network and the impact of serum vitamin D levels on the etiology, course, and severity of AIS.

Our findings clarify the dysregulation observed in the peripheral immune system linked to AIS and emphasize the possibility of developing novel therapeutic approaches for AIS by influencing the cytokine network and serum vitamin D concentrations. This could potentially target pathways involved in cytokine synthesis, such as NF-κB and JAK-STAT-1.

We used the CombiROC free online web tool, to assess the combined performance of cytokines and vitamin D signatures of stroke prognosis to support our hypothesis that immune-mediated inflammatory markers are indeed important in both pathogenesis and prognosis of AIS. Even though two other dual biomarker panels (IL-4- IFN-γ and IL-4- Vit D) were also selected as “gold combinations” on CombiROC, as diagnostic accuracy can be improved considerably by combining multiple markers [21], the triple-biomarker panel, IFN-γ, IL-4, and Vit D reporting the highest values for AUC, SE, and SP combinatorically was selected as the most accurate biomarker panel of AIS prognosis, suggestive of targeting inflammatory markers to be a promising therapeutic strategy in AIS. Further analysis using univariate binary logistic regression discerned that increased levels of IL-1β was associated with a poorer 30-day functional outcome while suggesting that increasing vitamin D levels may contribute to improved 30-day FO. However, no previous reports exist for the combinatorial accuracy of IFN-γ, IL-4, and vitamin D as a potential triple serum immune marker panel of stroke prognosis while reports of regression analysis of cytokines on AIS outcome are sparse. Furthermore, prognosis may be influenced by multiple other factors including the size and location of the infarct, patient comorbidities and timely appropriate intervention. These findings therefore require further validation with significantly larger numbers of subjects of similar cohorts. However, a binary logistic regression model fitted for functional outcome of AIS in our limited study indicated Th1:Th2 cytokine ratio (IFN-γ:IL-4), vitamin D status, history of stroke, and ischemic heart disease as significant predictors of AIS prognosis.

The outcomes of this study are exploratory due to the limited sample size. Furthermore, genetics, diet and medication were not explicitly addressed in this study which could also influence the concentrations of the tested serum immune mediators.

Novel treatment modalities for stroke are a critical need due to the narrow time window and lack of regenerative advantages that limit the potential of prevailing stroke treatment. Stem cell-based therapies may be a promising avenue due to their potential to address the unmet needs of stroke patients through neuroprotection and neuro-regeneration as well as expanding the window and availability of treatment while modulating the immune system along with mitigating the inflammatory damage [3,44]. Among the different stem cell types neural [45], bone marrow, mesenchymal, and umbilical cord stem cells have shown great potential to be effective in this venture[46,47]. Although still in need of exploration at both basic and translational levels, regenerative medicine using stem cells could be considered a panacea for post-stroke sequelae. Similarly targeting the assembly and activity of NLRP3 inflammasome activation could also pose as a potential therapeutic approach since this will reduce the maturation of proinflammatory cytokines such as IL-1β and IL-18 to prevent and/or alleviate AIS [48].

## Conclusions

Understanding the intricate role of inflammation in ischemic stroke pathogenesis is essential for developing targeted therapeutic strategies. Our study discerned that a Th1 cytokine polarized immune response predominated progression, while a Th2 cytokine-bias dominated the recovery phase of AIS. A low serum IL-1β and a high IL-4 level which were associated with a good functional outcome, suggest potential for therapeutic modulation of inflammatory cytokines in AIS. The poor prognosis of AIS associated with insufficient serum vitamin D levels in our study provides justification for further research on supplementation of vitamin D in AIS. IL-4– IFN-γ–Vit D may be proposed as a potential prognostic triple biomarker panel for AIS, which warrants further validation.

## Supporting information

S1 Fig Comparison of the performance of the prognostic marker panel (10 –fold cross validation [CV] and permutated model), generated using CV test for the best combos of prognostic markers. (PNG)

S1 Table Demographic and clinical data of the test and control subjects. (DOCX)

S2 Table Performance of the tested analytes as individual markers and as multiple biomarker panels for prognosis of acute ischemic stroke using CombiROC curve analysis. (DOCX)

S3 Table Comparison of the performance of the prognostic marker panel (10 –fold cross validation [CV] and permutated model), generated using CV test for the best combos of prognostic markers. (DOCX)

S1 Appendix Stepwise binary logistic regression model fitted for functional outcome of AIS. (DOCX)

S2 Appendix Minimal data set of the study. (XLSX)
