# Supplementary figures and images for: Selected serum cytokines and vitamin D levels as potential prognostic markers of acute ischemic stroke
Source: PLoS One. 2024 Jun 13;19(6):e0299631. doi: 10.1371/journal.pone.0299631 (PMC11175438; doi:10.1371/journal.pone.0299631)

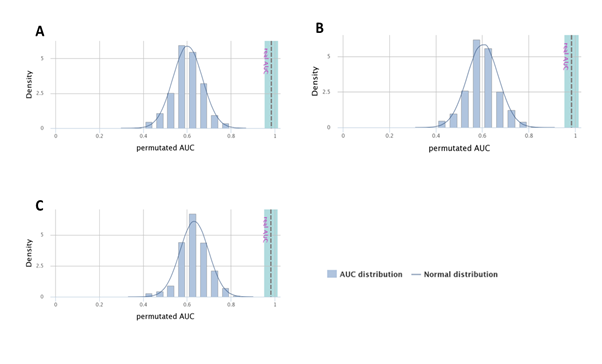

Supplement: S1 Fig — (PNG) [file pone.0299631.s001.png]
