# Supplementary material for: Selected serum cytokines and vitamin D levels as potential prognostic markers of acute ischemic stroke
Source: PLoS One. 2024 Jun 13;19(6):e0299631. doi: 10.1371/journal.pone.0299631 (PMC11175438; doi:10.1371/journal.pone.0299631)
Supplement: S1 Table — (DOCX) [file pone.0299631.s002.docx]

**S1 Table.** **Demographic and clinical data of the test and control subjects**

| **Study groups** | Mean age (years) (±SD) | | | | Gender  Male (%) | Mean NIHSS value on admission | Mean mRS score on 30 days follow up | p-value |
| --- | --- | --- | --- | --- | --- | --- | --- | --- |
| **Control group**  **(Normal healthy individuals**  **[n=15])** | | | 65 (±10.58) | 60 | | N/A | N/A | P >0.05 |
| Test groups  (Patients admitted post stroke onset [n=15/group]) | | | | | | | |  |
| **Group 1**  **(<6 h post AIS onset)** | | 56 (±19.03) | | | 73 | 23.33 (±5.97) | 2.26 (±0.88) | P>0.05 |
| **Group 2**  **(6-24 h post AIS onset)** | | 66 (±8.96) | | | 67 | 18.8 (±6.86) | 2.13 (±0.72) | P>0.05 |
| **Group 3**  **(24-48 h post AIS onset)** | | 68 (±10.49) | | | 67 | 22.27 (±8.19) | 1.93 (±0.83) | P>0.05 |
| **Group 4**  **(48-96 h post AIS onset)** | | 68 (±9.88) | | | 67 | 20.73 (±5.96) | 1.53 (±0.83) | P>0.05 |
