# Supplementary material for: Selected serum cytokines and vitamin D levels as potential prognostic markers of acute ischemic stroke
Source: PLoS One. 2024 Jun 13;19(6):e0299631. doi: 10.1371/journal.pone.0299631 (PMC11175438; doi:10.1371/journal.pone.0299631)
Supplement: S2 Table — (DOCX) [file pone.0299631.s003.docx]

**S2 Table. Performance of the tested analytes as individual markers and as multiple biomarker panels for prognosis of acute ischemic stroke using CombiROC curve analysis**

| **Marker/ Marker combination** | **AUC** | **SE** | **SP** | **Cutoff** |
| --- | --- | --- | --- | --- |
| Marker 1 | | | | |
| IL-1β | 0.979 | 0.913 | 0.973 | 0.683 |
| Marker 2 | | | | |
| IL-4 | 0.984 | 0.957 | 0.946 | 0.271 |
| Marker 3 | | | | |
| IFN-γ | 0.423 | 0.652 | 0.595 | 0.385 |
| Marker 4 | | | | |
| Vit D | 0.566 | 0.913 | 0.27 | 0.328 |
| Combo 1 | | | | |
| IL-1β -IL-4 | 0.986 | 1.000 | 0.865 | 0.152 |
| Combo 2 | | | | |
| IL-1β – IFN-γ | 0.976 | 0.913 | 0.973 | 0.651 |
| Combo 3 | | | | |
| IL-1β -Vit D | 0.978 | 0.913 | 0.946 | 0.65 |
| Combo 4 | | | | |
| IL-4- IFN-γ | **0.984** | **1.000** | **0.919** | 0.274 |
| Combo 5 | | | | |
| IL-4-Vit D | **0.984** | **1.000** | **0.919** | 0.232 |
| Combo 6 | | | | |
| IFN-γ -Vit D | 0.568 | 0.957 | 0.243 | 0.31 |
| Combo 7 | | | | |
| IL-1β -IL-4- IFN-γ | 0.986 | 0.957 | 0.919 | 0.416 |
| Combo 8 | | | | |
| IL-1β -IL-4-Vit D | 0.987 | 0.87 | 1.000 | 0.669 |
| Combo 9 | | | | |
| IL-1β – IFN-γ -Vit D | 0.974 | 0.913 | 0.946 | 0.612 |
| Combo 10 | | | | |
| IL-4- IFN-γ -Vit D | **0.982** | **1.000** | **0.919** | 0.272 |
| Combo 11 | | | | |
| IL-1β -IL-4- IFN-γ -Vit D | 0.988 | 0.913 | 0.973 | 0.581 |

AUC – area under the curve; SE- sensitivity; SP- specificity.
