# Supplementary material for: Selected serum cytokines and vitamin D levels as potential prognostic markers of acute ischemic stroke
Source: PLoS One. 2024 Jun 13;19(6):e0299631. doi: 10.1371/journal.pone.0299631 (PMC11175438; doi:10.1371/journal.pone.0299631)
Supplement: S3 Table — (DOCX) [file pone.0299631.s004.docx]

**S3 Table .** **Comparison of the performance of the prognostic marker panel (10 – fold cross validation [CV] and permutated model), generated using CV test for the best combos of prognostic markers**

| **Marker combination** | **Data set type** | **ACC** | **Error rate** | **SE (%)** | **SP** | **AUC** |
| --- | --- | --- | --- | --- | --- | --- |
| Combo 4 | | | | | | |
| IL-4- IFN-γ | 10–fold CV | 0.95 | 0.05 | 100 | 91.9 | 0.984 |
|  | Permutated model | 0.628 | 5.439 | 61.8 | 66.1 | 0.601 |
| Combo 5 | | | | | | |
| IL-4-Vit D | 10–fold CV | 0.95 | 0.05 | 100 | 91.9 | 0.984 |
|  | Permutated model | 0.627 | 5.235 | 61.2 | 67.1 | 0.601 |
| Combo 10 | | | | | | |
| IL-4- IFN-γ - Vit D | 10–fold CV | 0.95 | 0.05 | 100 | 91.9 | 0.982 |
|  | Permutated model | 0.645 | 5.172 | 63.4 | 68.1 | 0.632 |

ACC- Accuracy; SE – Sensitivity; SP – Specificity; AUC – Area Under the curve
