# Supplementary material for: Selected serum cytokines and vitamin D levels as potential prognostic markers of acute ischemic stroke
Source: PLoS One. 2024 Jun 13;19(6):e0299631. doi: 10.1371/journal.pone.0299631 (PMC11175438; doi:10.1371/journal.pone.0299631)
Supplement: S1 Appendix — (DOCX) [file pone.0299631.s005.docx]

**S1 Appendix. Stepwise binary logistic regression model fitted for functional outcome of acute ischemic stroke**

**Start: AIC=68.37**

mRS outcome ~ Cytokine_ratio + VitaminD + time_of_stroke_onset + history_of_stroke +

ischaemic_heart_disease + oxfordshire_stroke_classification + severity

| Variable | Df | Deviance | AIC |
| --- | --- | --- | --- |
| severity | 2 | 43.749 | 65.749 |
| Oxfordshire stroke classification | 3 | 45.931 | 65.931 |
| time_of_stroke_onset | 3 | 46.931 | 66.931 |
| <none> |  | 42.370 | 68.370 |
| C_ratio | 1 | 44.441 | 68.441 |
| history_of_stroke | 1 | 45.186 | 69.186 |
| ischaemic_heart_disease | 1 | 53.724 | 77.724 |
| VitaminD | 1 | 58.549 | 82.549 |

**Step: AIC=65.75**

mRS outcome ~ Cytokine_ratio + VitaminD + time_of_stroke_onset + history_of_stroke + ischaemic_heart_disease + Oxfordshire_stroke_classification

| Variable | Df | Deviance | AIC |
| --- | --- | --- | --- |
| time_of_stroke_onset | 3 | 48.330 | 64.330 |
| Oxfordshire stroke classification | 3 | 48.535 | 64.535 |
| <none> |  | 43.749 | 65.749 |
| Cytokine_ratio | 1 | 45.891 | 65.891 |
| history_of_stroke | 1 | 46.612 | 66.612 |
| ischaemic_heart_disease | 1 | 55.019 | 75.019 |
| VitaminD | 1 | 60.394 | 80.394 |

**Step: AIC=64.33**

mRS outcome ~ Cytokin e_ratio + VitaminD + history_of_stroke + ischaemic_heart_disease + Oxfordshire_stroke_classification

| Variable | Df | Deviance | AIC |
| --- | --- | --- | --- |
| Oxfordshire stroke classification | 3 | 51.606 | 61.606 |
| <none> |  | 48.330 | 64.330 |
| history_of_stroke | 1 | 52.052 | 66.052 |
| Cytokine_ratio | 1 | 53.274 | 67.274 |
| ischaemic_heart_disease | 1 | 57.617 | 71.617 |
| Vitamin D | 1 | 64.994 | 78.994 |

**Step: AIC=61.61**

mrsoutcome ~ C_ratio + VitaminD + history_of_stroke + ischaemic_heart_disease

| Variable | Df | Deviance | AIC |
| --- | --- | --- | --- |
| <none> |  | 51.606 | 61.606 |
| Cytokine_ratio | 1 | 54.627 | 62.627 |
| history_of_stroke | 1 | 57.128 | 65.128 |
| ischaemic_heart_disease | 1 | 60.439 | 68.439 |
| Vitamin D | 1 | 66.422 | 74.422 |

| Variable | Level | Estimate | Z-Value | p-Value |
| --- | --- | --- | --- | --- |
| Cytokine_ratio |  | **-0.1003** | **-1.683** | **0.0924** |
| Vitamin D status |  | **0.18197** | **2.698** | **0.00698** |
| Gender | 1 | -0.09271 | -0.162 | 0.872 |
| time_of_stroke_onset | **1** | **-0.5390** | **-0.730** | **0.466** |
|  | **2** | **0.8781** | **1.125** | **0.260** |
|  | **3** | **1.2528** | **1.514** | **0.130** |
| Age |  | 0.004726 | 0.184 | 0.854 |
| history_of_stroke | **1** | **-1.5939** | **-2.495** | **0.01260** |
| history_of_transient ischemic attack | 1 | 0.2877 | 0.459 | 0.646 |
| High_blood pressure | 1 | -0.2549 | -0.334 | 0.738 |
| Ischaemic_heart_disease | **1** | **-1.2277** | **-1.888** | **0.0590** |
| Cardiac_structural_abnormalities | 1 | 0.4951 | 0.561 | 0.575 |
| Atrial fibrillation | 1 | 0.2793 | 0.517 | 0.605 |
| Diabetes mellitus | 1 | -0.1029 | -0.149 | 0.882 |
| dyslipidemia | 1 | -0.2412 | -0.265 | 0.791 |
| family_history | 1 | 0.2288 | 0.182 | 0.8553 |
| Smoking |  | -0.03540 | -0.929 | 0.3528 |
| alcohol | 1 | 14.8729 | 0.010 | 0.9918 |
|  | 2 | -0.6931 | -1.115 | 0.2648 |
|  | 3 | -0.2877 | -0.387 | 0.6989 |
| Oxfordshire stroke classification | **1** | **0.7985** | **1.043** | **0.297** |
|  | **2** | **0.1054** | **0.071** | **0.944** |
|  | **3** | **0.9704** | **1.556** | **0.120** |
| Severity | **3** | **-0.5725** | **-0.749** | **0.4539** |
|  | **4** | **-1.1787** | **-1.700** | **0.0892** |
